# ﻿Descriptions of two new species associated with *Westermannia nobilis* Draudt, 1950 (Lepidoptera, Nolidae, Westermanniinae) from China

**DOI:** 10.3897/zookeys.1262.168662

**Published:** 2025-12-03

**Authors:** Yue Qin, Chao Zhang, Huilin Han

**Affiliations:** 1 School of Forestry, Northeast Forestry University, Harbin 150040, China Northeast Forestry University Harbin China; 2 Simianshan Forest Resource Service Center, Jiangjin District, Chongqing 402260, China Simianshan Forest Resource Service Center Chongqing China; 3 Northeast Asia Biodiversity Research Center, Northeast Forestry University, Harbin 150040, China Northeast Forestry University Harbin China; 4 Ministry of Education, Key Laboratory of Sustainable Forest Ecosystem Management, Northeast Forestry University, Harbin 150040, China Simianshan Forest Resource Service Center Chongqing China

**Keywords:** identification key, new species, nolid moths, taxonomy, *
Westermannia
*

## Abstract

Two new species, *Westermannia pseudonobilis* Qin & Han, **sp. nov.** and *W. simianshana* Qin, Zhang & Han, **sp. nov.**, which closely resemble *W. nobilis* Draudt, 1950 in both external appearance and genital characteristics, are described from China. Comparative diagnoses for these three species are provided, along with colour plates of adults and genitalia. An identification key and a distribution map are also included.

## ﻿Introduction

The genus *Westermannia* Hübner, [1821] 1816 is currently classified within the subfamily Westermanniinae of the family Nolidae (Zahiri et al. 2013). This highly diverse genus is widely distributed across subtropical and tropical regions of the Eastern Hemisphere. Its species typically have forewings with relatively broad outer margins and rounded tornal angles, and hindwings with full, rounded contours. According to Holloway (2003), the genus in its strict sense is defined by a set of highly conserved morphological features: a striking forewing pattern in silvery, glistening grey, brown, and black, which includes a medial figure-8 mark surrounded by paler colouration that extends to a distinctly angled or sinuous postmedial line.

In China, six species of this genus have been recorded: *W. superba* Hübner, 1823, *W. triangularis* Moore, 1877, *W. elliptica* Bryk, 1913, *W. antaplagica* Draudt, 1950, *W. jucunda* Draudt, 1950, and *W. nobilis* Draudt, 1950 (Bryk 1913; Draudt 1950; Chen 1999). Among these, the genitalia of three species: *W. elliptica*, *W. superba*, and *W. triangularis* have been described or illustrated in studies of adjacent regions (Sugi 1991; Kobes 1997; Holloway 2003). However, the genitalia of other species with distributions restricted to China remain poorly studied. This deficiency, with the relatively high diversity and morphological similarity within the genus, has resulted in significant research gaps and unresolved taxonomic issues.

*Westermannia nobilis* serves as a representative case of these taxonomic challenges. It was originally described by Draudt (1950) based on specimens collected from West Tianmu Mountain (= West-tien-mu-shan) in Zhejiang Province. Among the species distributed in China, it is morphologically similar to *W. elliptica* but can be distinguished by the configuration of the forewing postmedial line. In *W. elliptica*, this line is bent only in the upper portion (near R_5_ and M_1_ veins), whereas in *W. nobilis*, the lower portion (near M_3_ and Cu_1_ veins) also forms an outward-projecting angle (Figs 5–8), resulting in a more distinctly undulating postmedial line. *Westermannia nobilis* has been widely reported from eastern, central, and southern China (Chu et al. 1964; Chen 1999; Yang et al. 2017), as well as from Thailand (Kononenko and Pinratana 2013). However, these records are based primarily on external morphology.

To address the issues outlined above, we examined recent Chinese specimens identified as “*W. nobilis*” and discovered two new species that are externally highly similar to and share many genitalic characteristics with true *W. nobilis*, indicating a close relationship. In this paper, we describe these two new species as *W. pseudonobilis* Qin & Han, sp. nov. and *W. simianshana* Qin, Zhang & Han, sp. nov. We provide detailed illustrations of all three species, along with an identification key and a distribution map to better distinguish them. The discussion covers the limitations of our study and suggests directions for future research. All type specimens and additional material examined are deposited in the insect collection of Northeast Forestry University (**NEFU**), Harbin, China.

## ﻿Materials and methods

This study was based on the collection from the Insect Taxonomy Laboratory of the Northeast Forestry University. The method for preparing genitalia slides was based on Kononenko and Han (2007). Adults and genitalia were photographed using a Canon EOS 6D Mark II camera and an Olympus SZ61 stereomicroscope with camera port, and the photos were imported into Helicon Focus 7.6.6 software for depth-of-field focus stacking. The resulting images were subsequently processed using Adobe Photoshop 2020 and made into figures.

## ﻿Taxonomic account

### ﻿Genus Westermannia

Taxon classificationAnimaliaLepidopteraNolidae

Hübner, [1821] 1816

2EF496E5-4904-5BA5-88B1-B9A11BC13432

Westermannia Hübner, [1821] 1816, Verz. bek. Schmett.: 250. Type species: Westermannia superba Hübner, 1823, by original designation.
Plusiodes Guenée, 1852, in Boisduval and Guenée, Hist. nat. Ins., Spec. gén. Lépid. 5 (Noct. 1): 385. Type species: Plusiodes westermannii Guenée, 1852 (replacement of Westermannia superba Hübner, 1823).
Vestermannia Hampson, 1912, Cat. Lepid. Phalaenae Br. Mus. 11: 603 (emendation of Westermannia Hübner, [1821] 1816).

### ﻿ Westermannia pseudonobilis

Taxon classificationAnimaliaLepidopteraNolidae

Qin & Han sp. nov.

C9CCF29A-5A5C-5DC4-8C1F-DD64FEC08513

https://zoobank.org/62689112-2D3A-499B-A40E-E464EF598651

**Figures 1–8.**
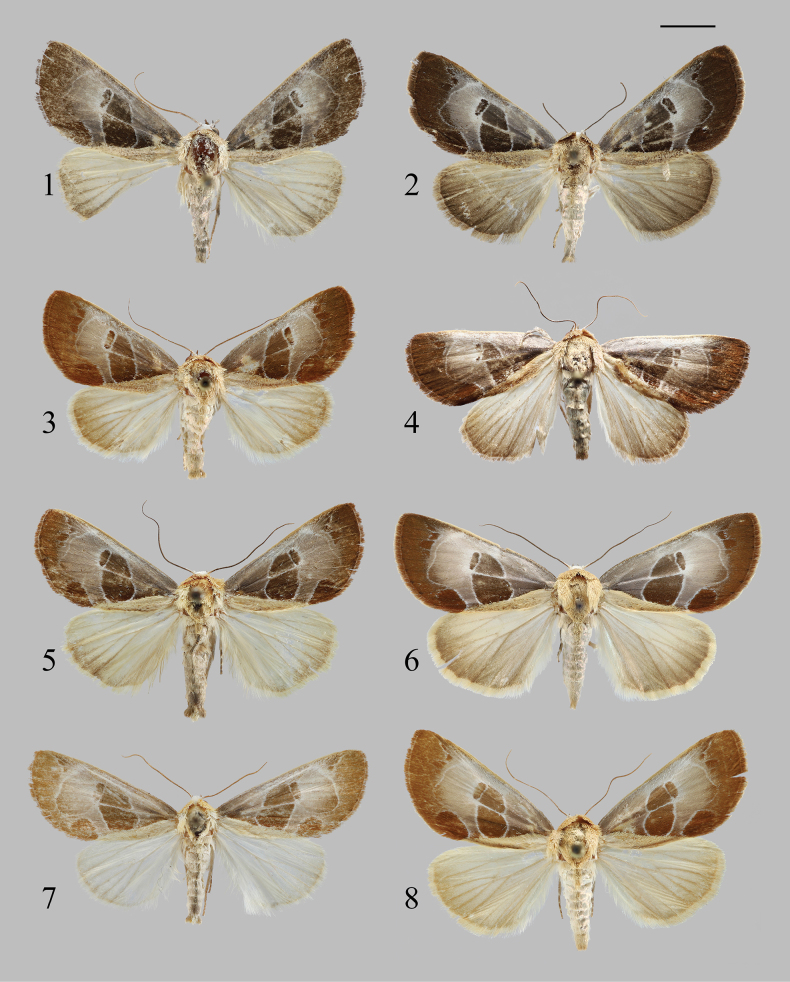
Adults of *Westermannia* spp. (1, 3, 5, 7: male; 2, 4, 6, 8: female). 1, 2. *W. pseudonobilis* Qin & Han, sp. nov., coll. NEFU (1: holotype; 2: paratype); 3, 4. *W. simianshana* Qin, Zhang & Han, sp. nov., coll. NEFU (3: holotype 4: paratype); 5–8 *W. nobilis* Draudt, 1950, collected from Guangxi (5), Hunan (6), and Chongqing (7–8), China, coll. NEFU. Scale bar: 5 mm.

**Figures 9–11.**
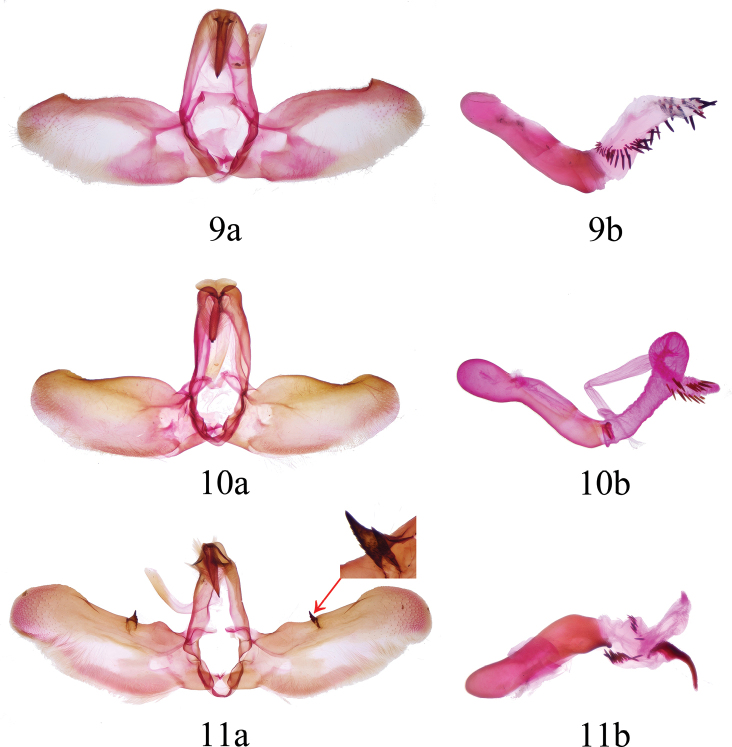
Male genitalia of *Westermannia* spp. (a: tegumen-vinculum; b: aedeagus). 9. *W. pseudonobilis* Qin & Han, sp. nov., genit. prep. QY-34, coll. NEFU; 10. *W. simianshana* Qin, Zhang & Han, sp. nov., genit. prep. QY-90 (a) and 36 (b), coll. NEFU; 11. *W. nobilis* Draudt, 1950 (with close-up of the processes on costa), genit. prep. QY-50 (51 for close-up), coll. NEFU

**Figures 12–14.**
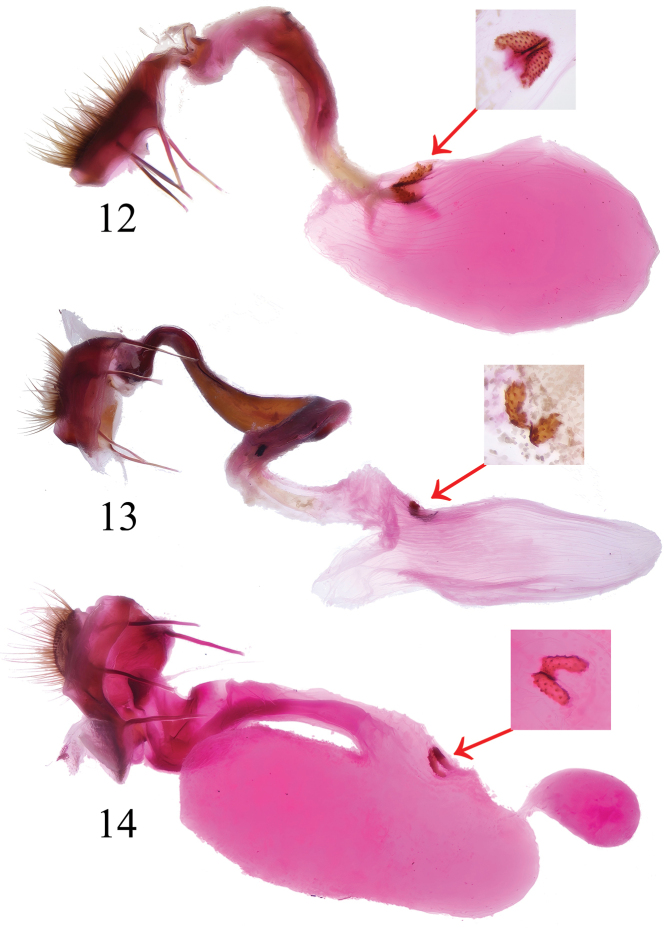
Female genitalia of *Westermannia* spp. with close-up of the signa. 12. *W. pseudonobilis* Qin & Han, sp. nov., genit. prep. QY-33 (75 for close-up), coll. NEFU; 13. *W. simianshana* Qin, Zhang & Han, sp. nov., genit. prep. QY-91 (45 for close-up), coll. NEFU; 14. *W. nobilis* Draudt, 1950, genit. prep. QY-52, coll. NEFU.

**Figure 15.**
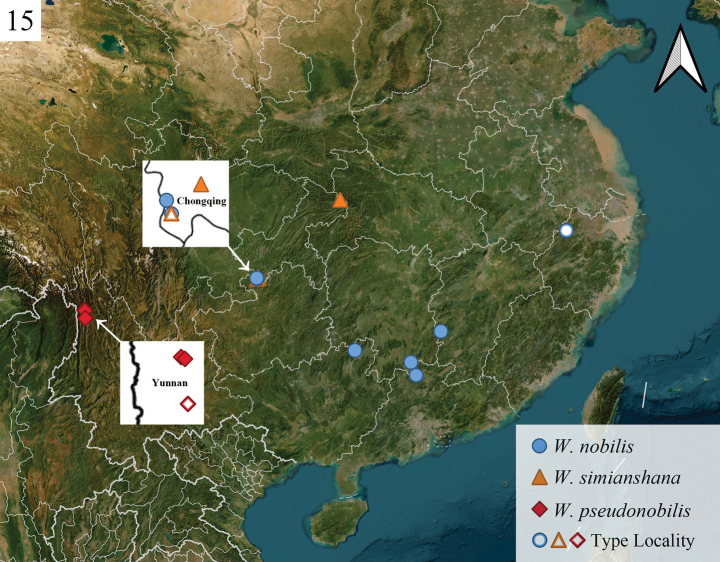
Distribution map and type localities of three *Westermannia* species in China.

Figs 1, 2, 9, 12, 15

#### Type material.

***Holotype***: • male, China, Yunnan, Weixi, Weideng Town, Xinhua Village, 29.VII.2024, leg. Y.Y. Jin, genit. prep. QY-34, NEFU. ***Paratypes***: • 2 females, China, Yunnan, Weixi, Baijixun Town, Nuoge Village, 30.VII.2024, leg. Y.Y. Jin, genit. prep. QY-33 and 75, NEFU • 1 female, CHINA, Yunnan, Weixi, Baijixun Town, Haizan Village, 2.VIII.2024, leg. Y.Y. Jin, genit. prep. QY-35, NEFU.

#### Diagnosis.

This species is closely related to *W. simianshana* Qin, Zhang & Han, sp. nov. and *W. nobilis* Draudt but differs as follows: generally darker in overall colour; the outwardly arcuate median portion of the postmedial line on the forewing is more acute. In the male genitalia, the sclerotized plate at the base of the uncus is smaller and elliptical; the valva is broader, with minute denticles along the costa; the vesica bears a spiral band of cornuti extending from base to apex. In the female genitalia, the ductus bursae has a sclerotized binding ring at the base, overall sinuous but untwisted, and the corpus bursae is ellipsoidal and lacking an appendix bursae. Additionally, this species has currently only been recorded in Yunnan Province.

#### Description.

**Adult** (Figs 1, 2). Wingspan 30.0–30.5 mm. ***Head*** mainly white. ***Antennae*** filiform, basal portion faintly whitish, remainder dark brown. ***Thorax*** with dorsum, tegula, and patagium khaki, densely pubescent. ***Abdomen*** greyish-white, apical portion greyish-brown, anal tuft khaki. ***Forewing*** silvery greyish-brown on costal, basal, and postmedian areas, gradually paler from inner margin to costal margin; median area and outer 1/3 dark brown; a longitudinal khaki band tinged with fuscous along inner margin, extending from base to tornal angle, upper margin straight; a 8-shaped filiform and pale lines in the median line area, Cu veins pale; reniform spot dark brown, wedge-shaped, foam pale, radiating bright white at the rear; postmedial line white, slender, strongly excurved at M_3_-Cu_2_; subterminal line represented by 4–5 faintly white-edged small spots near the costal margin; fringe dark brown, apically paler; terminal line and median line areas dark brown, other parts lighter. ***Hindwing*** predominantly greyish-white, outer margin 1/3 suffused with brown, females darker than males; venation distinct; fringe desert grey.

**Male genitalia** (Fig. 9). ***Uncus*** elongate, nail-shaped, acute apically, surrounded by brush of coarse setae, base covered with an oval sclerite. ***Tegumen*** narrowly lingulate, approximately twice the length of uncus. ***Vinculum*** narrow, V-shaped, saccus short, weakly developed. ***Juxta*** trumpet-shaped, weakly sclerotized. ***Valva*** broadly oblong, dorsal margin serrulate with minute teeth; ***cucullus*** rounded, scattered with distinct punctures; ***costa*** strip-shaped, basal 3/4 slightly raised, distal 1/4 with a prominent hill-shaped protrusion; ***sacculus*** relatively flat, basally slightly protruding outward, then weakly curved, densely covered with long hairs. ***Aedeagus*** cylindrical, slightly sigmoidally curved, basal 1/5 covered with granules; coecum swollen; carina moderately sclerotized. ***Vesica*** tubular, nearly equal in length to aedeagus, basal 2/3 similar in diameter to aedeagus, distal 1/3 gradually tapered and dorsally recurved; a helical band of cornuti originating from ventro-basal region, cornuti spine-like, heavily sclerotized, increasing in size and becoming sparser from base to apex.

**Female genitalia** (Fig. 12). ***Papillae anales*** broadly flattened, short, strip-shaped, densely setose apically. ***Apophysis anterior*** and ***posterior*** slender, subequal in length. ***Ostium bursae*** shallowly funnel-shaped. ***Ductus bursae*** sinuous, robust, chute-shaped in outline, moderately sclerotized at middle part, with a strongly sclerotized ring at base. ***Corpus bursae*** ellipsoidal, nearly equal in length to ductus bursae, surface wrinkled, bearing a pair of spiny-fusiform signa at ductus-corpus bursa junction.

#### Distribution.

China (Yunnan).

#### Etymology.

The specific epithet of this species is derived from that of the similar species *W. nobilis*, and because of its morphology similar to that of the latter, it is named *pseudo*-(meaning “false”) + *nobilis*.

### ﻿ Westermannia simianshana

Taxon classificationAnimaliaLepidopteraNolidae

Qin, Zhang & Han sp. nov.

27BC81AD-3E55-5A02-A6D3-05DC782FA1F5

https://zoobank.org/8F497A35-4F3B-412B-8444-C6D9C09DBF43

Figs 3, 4, 10, 13, 15

#### Type material.

***Holotype***: • male, China, Chongqing, Jiangjin, Simianshan, Dawopu, 12–13.VII.2018, leg. H.L. Han, genit. prep. QY-37, NEFU. ***Paratypes***: • 1 male, same locality as holotype, 29.VII–2.VIII.2020, leg. H.L. Han & J. Wu, genit. prep. QY-88, NEFU • 1 male and 2 females, same locality as for holotype, 23.VII–6.VIII.2018, leg. W.J. Li & G.X. Wang, genit. prep. QY-89, 90 and 91, NEFU • 1 male, China, Chongqing, Jiangjin, Simianshan, Tudiyan, 30.VII–3.VIII.2019, leg. T.T. Zhao & S.C. Deng, genit. prep. QY-76, NEFU • 2 females, China, Chongqing, Wuxi, Yintiaoling Reserve, Baiguo Station, 24–28.VI.2022, leg. T.T. Zhao & Y.Y. Jin, genit. prep. QY-45 and 46, NEFU.

#### Diagnosis.

This species is closely related to *W. pseudonobilis* Qin & Han, sp. nov. and *W. nobilis* Draudt but smaller in body size; the medial figure-8 mark on the forewing is relatively narrow; the colour of the two ends of the reniform spot is obviously darker; the patch on the tornal angle is not obvious; the outwardly arcuate median portion of the postmedial line is blunter. In male genitalia, the sclerotized plate at the base of the uncus is larger and lotus-leaf-shaped; the valva is smaller, with undulate margin but lacking additional processes; the coecum is markedly inflated; the vesica is slender, tubular, distally twisted, bearing two rows of cornuti. In female genitalia, the ductus bursae is elongate, anterior half strongly sclerotized and curved, posterior half membranous and helically twisted; the corpus bursae is fusiform, lacking an appendix bursae. Additionally, this species has currently only been recorded in Chongqing Municipality.

#### Description.

**Adult** (Figs 3, 4). Wingspan 30.0–32.5 mm. ***Head*** white. ***Antennae*** filiform, basal portion faintly whitish, remainder light brown. ***Thorax*** with dorsum, tegula, and patagium almond, densely pubescent. ***Abdomen*** whitish, apical portion and anal tuft almond. ***Forewing*** paler greyish-brown on costal area, basal area and postmedian area, gradually paler from inner to costal margin; median area and outer 1/3 chocolate-brown; a longitudinal almond band tinged with fuscous along the inner margin, extending from base to tornal angle, upper margin straight; a 8-shaped filiform and pale lines in the median line area, Cu veins pale; reniform spot dark brown, narrow strip-shaped, foam pale, radiating bright white at the rear; postmedial line white, slender, smooth excurved at M_3_-Cu_2_; subternimal line represented by 4–5 faintly white-edged small spots near costal margin; fringe chocolate-brown, apically paler; the terminal line and median line areas dark brown, other parts lighter. ***Hindwing*** predominantly whitish, outer margin faintly suffused with brown, females darker than males; venation distinct; fringe whitish, blended with brown.

**Male genitalia** (Fig. 10). ***Uncus*** elongate, nail-shaped, sharpened apically, surrounded by coarse setal brush, base overlain by a lotus leaf-shaped sclerite. ***Tegumen*** narrowly lingulate, approximately twice the length of uncus. ***Vinculum*** narrow, U-shaped, saccus short, weakly developed. ***Juxta*** trumpet-shaped, weakly sclerotized. ***Valva*** broadly oblong; ***cucullus*** rounded, scattered with distinct punctures; ***costa*** narrowly ribbon-shaped, smooth, round apically; ***sacculus*** basally inflected, then weakly curved, densely covered with long hairs. ***Aedeagus*** cylindrical, medially slightly curved, covered with faint granules at base; coecum swollen; carina weakly sclerotized. ***Vesica*** tubular, nearly equal in length to aedeagus, basal 2/3 similar in diameter to aedeagus, abruptly narrowed and twisted spirally in distal third; apical 1/4 slightly thickened, bearing two rows of spiniform, heavily sclerotized cornuti.

**Female genitalia** (Fig. 13). ***Papillae anales*** broadly flattened, short strip-shaped, densely setose apically. ***Apophysis anterior*** and ***apophysis posterior*** slender, subequal in length. ***Ostium bursae*** shallowly funnel-shaped. ***Ductus bursae*** sinuous, robust, with the anterior half strongly sclerotized and posterior half membranous, twisted into a helical shape. ***Corpus bursae*** fusiform, nearly equal in length with ductus bursae, surface wrinkled, bearing a pair of spiny-oblong signa at the ductus-bursa junction.

#### Distribution.

China (Chongqing).

#### Etymology.

The species name is derived from its type locality: Simianshan (= Mount Simian) in Chongqing Municipality, China.

### ﻿ Westermannia nobilis

Taxon classificationAnimaliaLepidopteraNolidae

Draudt, 1950

A70AE91D-9DB8-5335-B969-32546B8C059A

Figs 5–8, 11, 14, 15

Westermannia nobilis Draudt, 1950, Mitt. Münch. Ent. Ges. 40 (1): 150, pl. 9, fig. 11. Type locality: West-tien-mu-shan [Syntype: ZFMK (Blume et al. 2010)].
Westermannia nobilis : Chu et al. 1964: 70; Chen 1999: 857, pl. 39, fig. 28; Yang et al. 2017: 551, pl. 42, fig. 3.

#### Material examined.

China – **Jiangxi** • 1 female, Jinggangshan Scenic Spot, Huangyangjie Station, 2.VIII.2024, leg. H.L. Han & Haliya, genit. prep. QY-26, NEFU. – **Hunan** • 2 males and 1 female, Yizhang, Mangshan Reserve, Jiangjunzhai, 30.VII–7.VIII.2021, leg. J. Wu & Q Lin, genit. prep. QY-50, 51 and 25, NEFU. – **Guangdong** • 3 females, Nanling, 7–9.V.2011, leg. H.L. Han & Y.Q. Hu, genit. prep. QY-58, 59 and 60, NEFU • 2 females, Shaoguan, Ruyuan, Xiaohuangshan, viewing deck, 25.V.2021, leg. M.R. Li & G. Fu, genit. prep. QY-48 and 49, NEFU. – **Guangxi** • 1 male and 2 females, Ziyuan, Yinzhu Laoshan National Reserve, Shuangchahe Station, 17–18.VII.2021, leg. J.J. Fan & B. Gao, genit. prep. QY-47, 52 and 53, NEFU • 1 female, Maoershan, Jiuniutang, 19.IV.2002, leg. S.L. Hao & H.J. Xue, genit. prep. QY-43, NEFU. – **Chongqing** • 1 male and 1 female, Jiangjin, Simianshan, Dawopu, 23.VII–6.VIII.2018, leg. W.J. Li & G.X. Wang, genit. prep. QY-54 and 56, NEFU • 1 female, same locality as above, 12–13.VII.2018, leg. H.L. Han, genit. prep. QY-36, NEFU • 1 male and 1 female, same locality as above, 29.VII–2.VIII.2020, leg. H.L. Han & J. Wu, genit. prep. QY-55 and 57, NEFU • 1 male, Jiangjin, Simianshan Nature Reserve, 29.IV.2019, leg. Z.T. Wang & J.J. Fan, genit. prep. QY-85, NEFU • 1 male, Wuxi, Lanying Township, Xi’an Countryside, 26–28.VI.2022, leg. T.T. Zhao & Y.Y. Jin, genit. prep. QY-44, NEFU.

#### Distribution.

China (Zhejiang, Jiangxi, Hunan, Chongqing, Guangdong, Guangxi).

#### Remarks.

This study provides the first record of *W. nobilis* in Chongqing Municipality. This species is highly similar morphologically to the two newly described species in appearance, especially *W. simianshana* Qin, Zhang & Han, sp. nov., which is distributed in the same area as *W. nobilis* in Chongqing. Therefore, the geographical distribution of *W. nobilis* recorded in previous studies, for which we were unable to examine specimens, may require further dissections to be determined.

### ﻿Key to *Westermannia nobilis* and its two allied new species

**Table:** 

1	Forewing dark brown on outer 1/3; male valva with serrate costa; female ductus bursae with a sclerotized binding ring at the base	***W. pseudonobilis***
–	Forewing chocolate-brown on outer 1/3; male valva lacking serrations on costa; female ductus bursae without a sclerotized binding ring at the base	**2**
2	Tornal patch absent; male valva margin smooth, devoid of processes; female ductus bursae twisted, corpus bursae fusiform, appendix bursae absent	***W. simianshana***
–	Tornal patch present; male valva bearing two overlapped processes on the middle of costa; female ductus bursae widened at basal half, corpus bursae fabiform, with one elliptical appendix bursae	***W. nobilis***

## ﻿Discussion

Our study identifies three closely related species in the genus *Westermannia* that are highly similar in morphology and genitalia yet possess consistent and stable interspecific distinctions. This pattern of morphologically similar species complexes is not uncommon in the genus, as evidenced by Sugi’s (1991) informal concept of a “*superba* group” encompassing *W. elliptica* and *W. superba*. The characteristic high diversity and morphological conservatism within *Westermannia* underscore the necessity of establishing well-defined species groups to bring order to its taxonomy. Therefore, we suggest that a formal classification into species groups is a crucial next step for the systematics of this genus. Implementing this approach robustly, however, will require comprehensive global revision to establish rigorous definitions and boundaries for such groups.

Furthermore, the genus *Westermannia* also presents certain taxonomic issues at the generic level. Holloway (2003) noted that the relationships among African species currently placed in this genus, the species of *Miaromima* Meyrick, 1889, and those strictly defined within *Westermannia* remain unresolved and require further investigation. Additionally, the monotypic Eastern Asian genus *Iragaodes* Matsumura, 1931, which shares certain morphological and genitalic similarities with *Westermannia*, must be incorporated into any comprehensive revision. In fact, research of major lineages of Nolidae conducted by Zahiri et al. (2013) included these three genera and confirmed that they form a distinct clade within the subfamily Westermanniinae. However, their analysis was limited by a small number of sampled species. Although genetic data are currently unavailable for *W. nobilis* and two newly described species, we emphasize that molecular phylogenetic studies of this genus are imperative.

## Supplementary Material

XML Treatment for Westermannia

XML Treatment for Westermannia pseudonobilis

XML Treatment for Westermannia simianshana

XML Treatment for Westermannia nobilis

## ﻿Additional information

### Use of AI

No use of AI was reported.

### Author ORCIDs

Yue Qin https://orcid.org/0009-0009-3399-8560

Chao Zhang https://orcid.org/0000-0001-6069-1059

Huilin Han https://orcid.org/0000-0002-2045-6182

### Data availability

All of the data that support the findings of this study are available in the main text.
